# Using Deep Neural Networks to Improve Contact Wrench Estimation of Serial Robotic Manipulators in Static Tasks

**DOI:** 10.3389/frobt.2022.892916

**Published:** 2022-04-28

**Authors:** Jonas Osburg, Ivo Kuhlemann, Jannis Hagenah, Floris Ernst

**Affiliations:** ^1^ Institute for Robotics and Cognitive Systems, University of Lübeck, Lübeck, Germany; ^2^ Department of Engineering Science, University of Oxford, Oxford, United Kingdom

**Keywords:** deep learning, force estimation, wrench estimation, robotic manipulator, artificial neural network

## Abstract

Reliable force-driven robot-interaction requires precise contact wrench measurements. In most robot systems these measurements are severely incorrect and in most manipulation tasks expensive additional force sensors are installed. We follow a learning approach to train the dependencies between joint torques and end-effector contact wrenches. We used a redundant serial light-weight manipulator (KUKA iiwa 7 R800) with integrated force estimation based on the joint torques measured in each of the robot’s seven axes. Firstly, a simulated dataset is created to let a feed-forward net learn the relationship between end-effector contact wrenches and joint torques for a static case. Secondly, an extensive real training dataset was acquired with 330,000 randomized robot positions and end-effector contact wrenches and used for retraining the simulated trained feed-forward net. We can show that the wrench prediction error could be reduced by around 57% for the forces compared to the manufacturer’s proprietary force estimation model. In addition, we show that the number of high outliers can be reduced substantially. Furthermore we prove that the approach could be also transferred to another robot (KUKA iiwa 14 R820) with reasonable prediction accuracy and without the need of acquiring new robot specific data.

## 1 Introduction

### 1.1 Motivation

Compliant robotic arms have increasingly gained importance during the last years, where anthropomorphic kinematically redundant serial manipulators with seven degrees of freedom (DoF) are frequently used for various new applications. Integrated joint torque sensors provide crucial functionalities for safe human-robot-interactions. Based on the joint torques *τ*, measured in each of the robot’s axes, the corresponding contact forces f applied to the robot’s end-effector can be calculated. Obviously, a higher accuracy of the determined forces leads to enhanced overall sensitivity of the robot, and therefore allows for more complex applications. On the other hand, errors in the computational model can lead to dangerous and harmful situations during force driven operations. For industrial manipulators, no individual but rather general mechanical models are used for the control algorithms. Manufacturing inaccuracies and individual characteristics of bearings, sensors and actuators are often neglected. As a result, this leads to further sources of error in contact force determination and can have a strong influence on the resulting accuracy. In a dynamic scenario with high accelerations and velocities, mass induced inertial, centrifugal and Coriolis forces at the manipulator must be considered in the control algorithms Khatib (1987). For a static scenario the determination of end-effector contact forces is greatly simplified. Finally, only the inverted geometric *Jacobian Matrix* is required to express the relationship between forces and joint torques Schweikard and Ernst (2015). Whenever computational models use the *Jacobian Matrix* for motion and force control, the matrix loses full-rank at singularities, causing the computation of contact forces to fail Maciejewski (1991).

Furthermore, robots are usually not used in their full performance range, but operate in a very small range of forces due to the rather restricted type of specialized applications. Therefore, an optimized calibration within this specific range of forces could lead to very precise results, minimized errors and drastically increased overall system accuracy.

One approach to increase the accuracy of the contact force calculation is machine learning. Artificial neural networks show impressive capabilities in solving direct Sadjadian et al. (2005) and inverse kinematics Raj et al. (2015) as well as in dynamic control of redundant manipulators Kumar et al. (2011a), Lian (2014), Li et al. (2017). Earlier research addressed the calibration of force/torque sensors for serial robotic manipulators, mostly focusing on optimizing linear relations between joint torques and dynamic parameters known as the Inverse Dynamic Identification Model (IDIM) Khosla and Kanade (1985), Hollerbach et al. (2008), Gautier and Jubien (2014). Learning approaches to optimize the IDIM were presented in Kumar et al. (2011b), Pei et al. (1992), Jung et al. (2001). In Lu et al. (1997), neural networks are used to map measured signals and resulting forces, to calibrate an external force/torque sensor mounted to an end-effector. Although the authors show accurate results and provide time-saving routines, unfortunately, instabilities near singularities still exist or additional external sensors are required. Smith et al. showed interesting results by using a neural-network-based approach to determine contact forces for haptic devices Smith et al. (2006). Unfortunately, the authors have to use biased ground truth data and, therefore, it remains unclear how this affects the calibration accuracy.

An approach using deep learning for reducing errors in identification of dynamic parameters of a 6-DoF robot is presented in Wang et al. (2020). Moreover Lutter et al. (2019) include physical laws of the system (in form of the Euler-Lagrange equation) into deep neural network architectures. Thus more accurate models can be obtained whilst ensuring physical plausibility. However the papers do not relate to force estimation at the end-effector. This is done in Kružić et al. (2021), where deep learning is used to estimate end-effector forces and joint torques of a 7-DoF robotic manipulator. Even though promising results are shown, a validation of the estimation end-effector forces and moments on a real robot is missing.

The use of neural networks for calibrating the robotic system provides several advantages. The system is capable of learning unique mechanical characteristics of the manipulator and the robot can therefore be calibrated in a highly specialized way. Furthermore, critical arm positions and singularities can be directly learned from the network as training points. Using a sufficient amount of diverse input data, these points can be uniquely identified and integrated into the model, resulting in a more robust calibration.

### 1.2 Contribution of This Paper

The aim of this work is to improve the accuracy of static end-effector contact wrench estimation using the robot’s integrated sensory technology. A scenario with comparatively small contact forces (up to 20 N) well below the maximal capacity of the robot and small distances to the force application point in the tool (up to 0.15 m) is chosen. This scenario represents our use case holding an ultrasound probe, which is attached to the end-effector, in safe contact with the body. Even though this task can be considered quasi-static since the robot may move slightly, the dynamic effects due to the movement are very small compared with the gravitational forces as well as the external force exerted by the human body being in contact with. Thus, the movement can be seen as a point-to-point motion, with a wrench estimation being taken at each point while the robot is not moving and therefore is in a static state at the time of measurement.

#### 1.2.1 Contact Wrench Generation

Standard approaches for acquiring ground truth data for contact wrenches by using force/torque sensors or additional collaborating manipulators require expensive hardware and introduce various sources of errors due to mechanical issues. We present an alternative method to generate contact wrenches by mounting calibration loads to the end-effector. By using the gravity force in combination with different robot base orientations, we obtain a homogeneous representation of contact forces in all directions. An extensive database consisting of 330,000 randomized data points was created.

#### 1.2.2 KUKA Iiwa Accuracy Analysis

Our database allows for a detailed analysis of the robot’s integrated sensors and the accuracy of the proprietary force estimation model (PFEM).

#### 1.2.3 Gravity Torque Estimation

We present a data-driven method based on linear regression to approximate the static gravity torques without any knowledge of the link masses nor the centers of gravity of the links. The approach is easy to implement, data-saving and performs slightly better than the robots integrated estimation of gravity torques in a static case.

#### 1.2.4 Wrench Estimation Error Reduction

We follow a learning approach to train deep feed-forward artificial neural networks (ANNs) with simulated created as well as real data to estimate the contact wrenches applied to the end-effector based on the measured joint torques and information about the robot’s current pose. Estimation error and robustness close to singular joint configurations will be improved. Moreover we show that the approach could be transferred to another, similar robot (*KUKA LBR iiwa 14 R820*) with reasonable performance.

## 2 Materials and Methods

We used a *KUKA LBR iiwa 7 R800* robot for our experiments and data acquisition. However, the methods described in this paper are applicable to general serial kinematics equipped with joint torque sensors.

### 2.1 Robotic Manipulator

**FIGURE 1:**
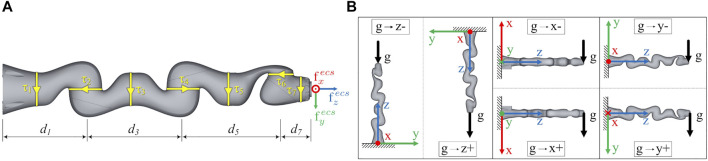
**(A)** The end-effector contact wrenches ${\text{f}}_{x}^{\;ecs}$ , ${\text{f}}_{y}^{\;ecs}$ and ${\text{f}}_{z}^{\;ecs}$ are related to the measured joint torques *τ*_1_, … , *τ*_7_. **(B)** The robot was mounted in six different base orientations to record the training data with a homogeneous distribution of gravity vector directions.

*KUKA’s LBR iiwa 7 R800* is a kinematically redundant serial light-weight manipulator with integrated joint torque sensors Bischoff et al. (2010). The robot has an additional 7^*th*^ joint, allowing for motion of the elbow on a circular path. The kinematic structure, related joint torques and end-effector contact wrenches are shown in Figure 1A. The manipulator provides seven DoF and has an S-R-S (spherical-rotational-spherical) kinematic structure. Different types of torque sensors are integrated in the axis of the robot. The sensors in the first two joints have a measuring range of ±176 Nm with a resolution of 1.344e-3 Nm. The sensors in joint 3-5 cover a range of ±110 Nm with a resolution of 0.88e-3 Nm and the sensors in joint 6 and 7 measure torques in the range of ±40 Nm with a resolution of 0.334e-3 Nm. The axis specific relative measuring error is 2%.

### 2.2 Proprietary Force Estimation Model

A detailed description of the proprietary robot control architecture is given in Albu-Schäffer et al. (2007), Haddadin et al. (2017). For the sake of clarity, the following section discusses essential force control formulations and points out how the proprietary model calculates end-effector contact forces. In the presence of external joint torques due to contact forces, the following dynamics model of robots with flexible joints is considered:

$$M\left(\text{q}\right)\ddot{\text{q}}+C\left(\text{q},\dot{\text{q}}\right)\dot{\text{q}}+\text{g}\left(\text{q}\right)=\tau_{J}+\tau_{\text{ext}}.$$ (1)

$M\left(\text{q}\right)\in R^{n\times n}$ is the symmetric and positive-definite inertia matrix, $C\left(\text{q},\dot{\text{q}}\right)\in R^{n}$ is the centrifugal and Coriolis matrix, and $\text{g}\left(\text{q}\right)\in R^{n}$ is the gravity vector. We denote $\tau_{\text{ext}}\in R^{n}$ as the external joint torque and $\tau_{J}\in R^{n}$ is the elastic torque transmitted through the joints as *τ*_*J*_ = **K**_*J*_ (*θ* − q) where $K_{J}\in R^{n\times n}$ is the diagonal and positive definite joint stiffness matrix and $\theta \in R^{n\times n}$ are the motor positions. Let ${\text{F}}^{\;ecs}\in R^{6}$ be the wrench at the end-effector as

$${\text{F}}^{\;ecs}=\left[\begin{array}{c} {\text{f}}^{\;ecs}\\ {\text{m}}^{\;ecs} \end{array}\right]$$ (2)

consisting of contact forces ${\text{f}}^{\;ecs}\in R^{3}$ and moments ${\text{m}}^{\;ecs}\in R^{3}$ . By using the transposed geometric Jacobian matrix **J**(q) of the end-effector we can calculate the external joint torques as

$$\tau_{\text{ext}}=J^{\text{T}}\left(\text{q}\right){\text{F}}^{\;ecs}.$$ (3)

Finally, by including (3) in the general robot dynamics from (1) we can calculate the end-effector contact forces as

$${\text{F}}^{\;ecs}={J^{\text{T}}\left(\text{q}\right)}^{-1}\left(M\left(\text{q}\right)\ddot{\text{q}}+C\left(\text{q},\dot{\text{q}}\right)\dot{\text{q}}+\text{g}\left(\text{q}\right)-\tau_{J}\right).$$ (4)

### 2.3 Acquisition of Datasets

The aim of this work is to determine contact wrenches at the end-effector. It is investigated whether the contact wrench can be predicted from corresponding joint positions as well as joint torque data. Different datasets, both simulated and real have been acquired to train and evaluate neural network models.

#### 2.3.1 Simulated Training Data

A large simulated dataset is generated, varying the applied forces, the distances of the end-effector to the force application point as well as the applied external torques for randomized joint configurations. Thus, randomized end-effector contact wrenches are generated and the corresponding joint torques for an ideally static case can be calculated. In our scenario forces between −20 and 20 N are applied at a point with a distance between 0 and 0.15 m to the end-effector. The range results from our application to measure the force acting on an ultrasonic probe attached to the end-effector of a robot. Moreover an additional external torque between −2 and 2 Nm is applied at the end-effector. The steps of generating the simulated training data are explained as follows: Let ${\text{F}}^{\;ecs}\in R^{6}$ be the wrench at the end-effector consisting of contact forces ${\text{f}}^{\;ecs}\in R^{3}$ and moments ${\text{m}}^{\;ecs}\in R^{3}$ according to (2). For each data point a valid joint configuration, a contact force ${\text{f}}^{\;ecs}\in R^{3}$ and distance r^*fap*^ to the force application point as well as an additional external contact torque m^*ecs*,*ext*^ are chosen randomly. The resulting moment m^*ecs*^ applied at the end-effector can be calculated by

$${\text{m}}^{\;ecs}={\text{r}}^{\;fap}\times {\text{f}}^{\;ecs}+{\text{m}}^{\;ecs,ext}.$$ (5)

By using the transposed geometric Jacobian matrix **J**(q) of the end-effector we can calculate the corresponding external joint torques according to (3) considering an ideally static case. The simulated dataset consisting of 3,000,000 data points is named *dataset*_*train*,*sim*_.

#### 2.3.2 Real Training Data

**FIGURE 2:**
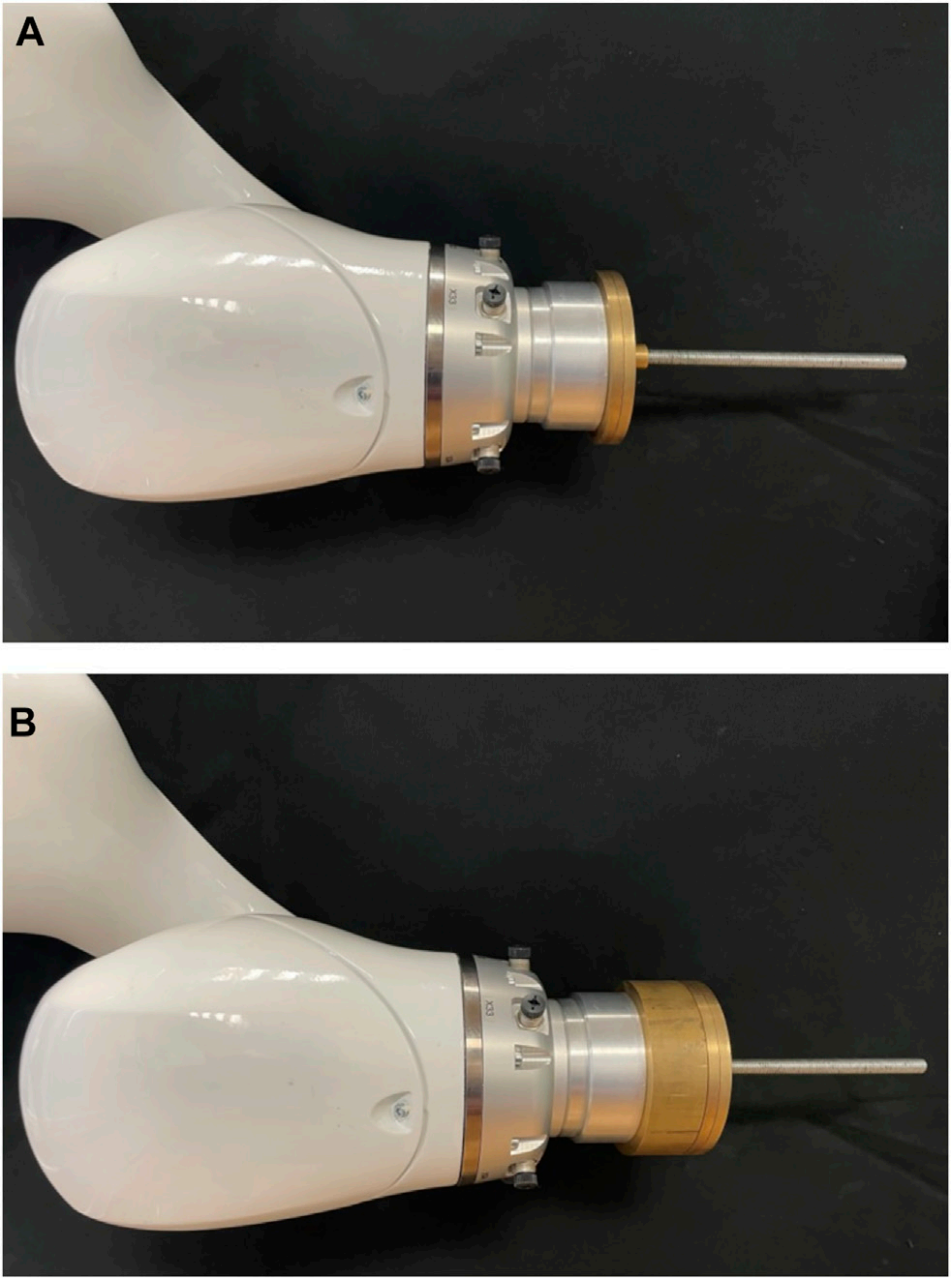
Calibration weights were stacked on a metallic rod attached to the end-effector of the robot to generate 10 equidistantly distributed end-effector forces in our target range from 0–20 N. Examples of appropriately stacked calibration weights to generate an end-effector force of 8 N **(A)** and 18 N **(B)** are shown.

The aim of this work is to precisely determine corresponding contact wrenches at the end-effector from given joint positions and torque data. Contact wrenches at the end-effector can result from various impacts: by external forces such as pushing or pulling by hand, or while the robot actively pushes against an object with its tool attached to the end-effector. From a mechanical point of view, the resulting wrenches are the same for both cases and can be measured via the joint torques. In this study, the wrenches are simulated by mounting different masses on the end-effector. Gravity produces an equivalent force, which pulls the mass towards the ground. Obviously, this static force will always point in the same direction in the world coordinate system, but for varying robot positions it will create different contact forces in end-effector coordinates. With only one direction in world coordinates not all contact forces can be represented. Hence, six different base rotations were used to solve this problem. The robot was therefore mounted in different orientations, shown in Figure 1B, resulting in a more homogeneous representation of the contact forces in all directions. To determine an exact ground truth for the end-effector forces, specially manufactured calibration weights were used. The weights are built from symmetric metallic discs. By appropriately stacking combinations of these calibration weights on a metallic rod, which was attached to the robots tool flange along the *z*-axis, we were able to generate 10 equidistantly distributed end-effector forces in 2 N steps in our target range from 0–20 N. The precise total weights resulting from the combinations of stacked calibration weights are *m*_*j*_ ∈ {0, 202, 391, 616, 818, 995, 1199, 1401, 1603, 1808, 2002} g, for *j* ∈ {1 … 11}. Examples of how the calibration weights are stacked on the metallic rod to generate specific end-effector forces are shown in Figure 2. For each mass, 5,000 measurements in newly randomly generated poses were acquired. Thereby sensor hysteresis is part of the data since we approach a multitude of different poses with different combinations of approach directions for each joint. In total a dataset of randomized 330,000 data points with varying base rotations and calibration weights was acquired. The force application point is chosen as the resulting centre of mass of the metallic rod with the appropriately stacked calibration weights. The centers of mass of the corresponding combinations of the metallic rod and the calibration weights are *com*_*j*_ ∈ {0, 2.5, 25.5, 28.2, 30.9, 33.4, 38.2, 40.7, 43.2, 42.3, 50} mm, for *j* ∈ {1 … 11}. These were determined from an accordingly created CAD model in SolidWorks, where the different materials as well as their densities were taken into account.

The exact contact forces for the ground truth can be determined as follows:

For a serial manipulator we can compute the position and orientation of the end-effector by coordinate transformations from the base along the joints by

M70=T10T21T32…T76. (6)

Using (6) we can also compute the transformation Mi0 , *i* ∈ {1 … 7} to every joint *i*.

Let M0,i−1 be the robot base transformation defined by

M0,i−1=Rz,ΨiRy,ΘiRx,Φi (7)

using homogeneous rotations with six different combinations of the three Euler angles Ψ_*i*_, Θ_*i*_ and Φ_*i*_, *i* ∈ {1 … 6}, denoted in Table 1. Let $g_{0}=9.806\frac{m}{s^{2}}$ be the gravity constant. the acceleration vector due to gravity is

$$g={\left[\begin{array}{ccc} 0 & 0 & -g_{0} \end{array}\right]}^{T}$$ (8)

Then the forces induced by the calibration masses m_*j*_ acting in the robot base coordinate system (*bcs*) can be described by

fi,jbcs=M0,i−1g⋅mj1 (9)

for *i* ∈ {1 … 6} and *j* ∈ {1 … 11}. Now let R70 be the end-effector rotation as upper 3 × 3 rotational matrix based on the homogeneous transformation from (6), i.e.

M70=R70t01 (10)

with vector t as translational part. We denote

R70=R70001 (11)

as the end-effector rotation as homogeneous transformation with zero translation in the robot base coordinate frame. Then the forces acting in the end-effector coordinate system (*ecs*) can be described by

fi,j,necs=R7n−10⋅fi,jbcs (12)

for *i* ∈ {1 … 6} base rotations, *j* ∈ {1 … 11} calibration masses and *n* ∈ {1 … 5,000} measurements.

**TABLE 1:** To calculate the six base transformations M0,i−1 using (7) as shown in Figure 1B, different combinations of Euler angles are used.

i	Base rotation	Ψ [°]	Θ [°]	Φ [°]
1	g → z −	0	0	0
2	g → z +	0	0	180
3	g → x −	180	−90	0
4	g → x +	0	90	0
5	g → y −	90	0	90
6	g → y +	−90	0	−90

The forces are not acting directly in the end-effector coordinate system, but in the centre of mass of the metallic rod with the appropriately stacked calibration weights. Thus, a moment m^*ecs*^ is generated in the end-effector, which can be calculated by

$${\text{m}}_{i,j,n}^{\;ecs}=r_{j,n}^{\;com}\times f_{i,j,n}^{\;ecs}.$$ (13)

For every end-effector wrench ${\text{w}}_{i,j,n}^{\;ecs}={\left[f_{i,j,n}^{\;ecs}\;m_{i,j,n}^{\;ecs}\right]}^{T}$ , the corresponding joint positions q_*i*,*j*,*n*_ and joint torques *τ*_*i*,*j*,*n*_ are measured using the robot control software *KUKA Sunrise* with the *Fast Research Interface (FRI) v1.13* (Schreiber et al., 2010). However, the measured torques include the self-weight of the robot links. The resulting torques depend on the joint positions and, more importantly, on the base orientation and the inherent direction of gravity. To decouple the data, only the torques generated by the specific calibration weights are used for training the neural network. To identify these isolated torques, the gravity torques without mounted calibration weight *τ*_0_ (m_0_) have to be estimated first (see Section 2.4) and subtracted from the measured joint torques with mounted calibration weight *τ*_*j*_ (m_*j*_). The torques resulting only from the additional weight can therefore be determined by

$$\tau_{j}^{\Delta }=\tau_{j}-\tau_{0}$$ (14)

for *j* ∈ {1 … 11} calibration weights. To have a database for the estimation of the gravity torques as well (see Section 2.4), two discrete measurements were performed at each of the randomized positions. First the joint torques without mounted calibration weight *τ*_0_ (m_0_) were recorded. In a second measurement, the torques with mounted calibration *τ*_*j*_ (m_*j*_) were recorded at the very same positions. Collecting the training data required approximately 11 weeks of continuous operation of the robot. This large training dataset consisting of 330,000 randomized positions is named *dataset*_*train*, *real*_.

#### 2.3.3 Real Generalisation Testing Data

**FIGURE 3:**
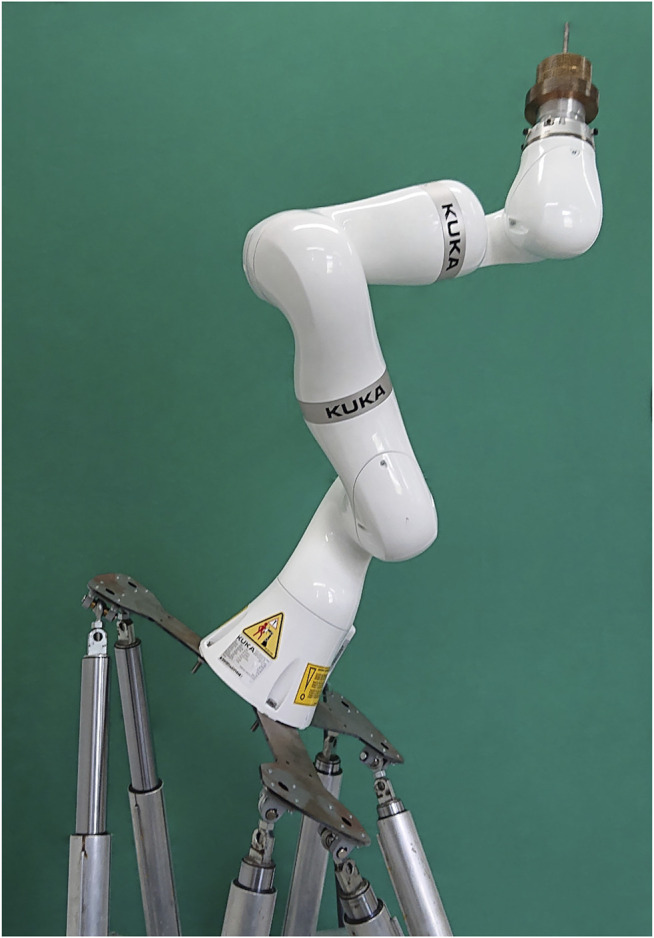
The LBR iiwa was mounted on a hexapod to acquire the test data (*dataset*_*gen*,*dir*_), to evaluate the intra-directional generalization performance.

As described in Section 2.3.2, six different base orientations are used to generate combinations of contact forces. In a realistic scenario, however, these end-effector forces can occur from every direction. Thus, the network must be capable of generalizing intra-directionally. To analyze the directional generalization performance, an additional test dataset was acquired using five varying, unleveled base orientations (see Table 2). Therefore, the manipulator was mounted on a hexapod, as shown in Figure 3. This dataset is only used for evaluating the performance of our models (see Figures 5, 6 in Chapter 3.2)–it is not involved in any form during the training process. Due to the lack of absolute precision of the hexapod, the actually reached Euler angles, for calculating the base orientation (see Section 2.3.3), were measured using an inclinometer with an accuracy of 0.1°. We used a calibration mass of 1.0 kg (10 N). The first dataset was acquired with a known base orientation (zero rotation) as reference. Afterwards the measurements for each orientation were acquired. The dataset is named *dataset*_*gen*,*dir*_.

**TABLE 2:** Base orientations of directional generalization *dataset*_*gen*,*dir*_.

Data points	Ψ [°]	Θ [°]	Φ [°]	Load [kg]
1,500	0.0	0.2	0.1	1.0
500	0.0	0.0	31.1	1.0
1,500	0.5	29.1	0.2	1.0
1,500	0.0	27.8	−14.6	1.0
1,500	0.0	−43.3	0.7	1.0
1,500	0.0	−31.7	39.4	1.0

#### 2.3.4 Real Transfer Testing Data

Generating a large robot specific training dataset as described in Section 2.3.2 is quite time-consuming. To examine the transferability of our approach to another robot type, an additional testing dataset was acquired. This dataset is only used for evaluation purposes (see Figure 7 in Section 3.4), it is not involved during the training process of the neural networks. This transfer testing dataset was acquired with the *KUKA iiwa 14 R820*. This robot has similar geometric dimensions and thus is well suited for a first experiment/trial. Due to the increased time required as well as the significantly larger self-weight of this robot, which does not allow mounting on our lab wall or lab ceiling, we only generated a dataset in the ground base orientation *g* → *z* − (see Figure 1B). For each calibration weight (0.2–20 N) 500 randomized positions are recorded in the same way as described in Section 2.3.2. This results in a dataset of 5,000 positions, which is named *dataset*_*transf*_. The gravity torques *τ*_0_ (m_0_) of the *KUKA iiwa 14 R820* are greater than for the smaller *KUKA iiwa 7 R800* since the robot links are bigger and heavier. Thus the gravity compensation parameters according to Section 2.4 have to be redetermined for the *KUKA iiwa 14 R820*, which is done in the same way as described in Section 3.1 using the *dataset*_*transf*_.

### 2.4 Gravity Torque Compensation Model

For training of the neural network the isolated torques $tau_{j}^{delta}$ generated by the specific calibration weight are used. To calculate them according to (14), the joint torques without mounted calibration weight *τ*_0_ have to be estimated first. In a static case these torques *τ*_0_ depend on the base orientation, the joint configuration and the weight of the robot links. We use a simple data-driven approach based on linear regression, which can be used without any knowledge of the masses as well as the centers of gravity of the robot links. Yu et al. (2019) showed that the joint angle and joint driving torque can accurately regress the relationship between barycentric coordinates of the link and the mass of the connecting rod in the Cartesian coordinate system. But instead of identifying the masses and centers of gravity of the robot links separately, we directly calculate parameters *w*_*j*_ by linear regression, which can be used to approximate the acting gravity torques due to the following joint link masses. Our approach is based on the following mechanical relation:

For a 7-DOF manipulator the relationship between a joint torque *τ*_*j*_ and the gravity forces *G*_*i*_ acting in the centres of the following links can be shown to be

$$\tau_{j}=\overset{7}{\underset{i=j}{\sum }}J_{m,i}^{T}\cdot G_{i}$$ (15)

where

$$\begin{array}{ll} J_{m,i}^{T} & ={\left[z_{j}\times r_{com,i}\right]}^{T}\\ & ={\left[\begin{array}{c} J_{m,x,i}^{T}\\ J_{m,y,i}^{T}\\ J_{m,z,i}^{T} \end{array}\right]}^{T}\\ & ={\left[\begin{array}{c} z_{j,y}r_{com,i,z}-z_{j,z}r_{com,i,y}\\ z_{j,x}r_{com,i,z}-z_{j,z}r_{com,i,x}\\ z_{j,x}r_{com,i,y}-z_{j,y}r_{com,i,x} \end{array}\right]}^{T} \end{array}$$ (16)

is the linear velocity part of the transposed jacobian for the centre of mass *r*_*com*_, *i* of link i. Since we are calculating in the world coordinate system, the force *G*_*i*_, acting in the centre of mass of link *i* due to the gravity of *m*_*i*_, is described by

$$g_{i}=\left[\begin{array}{c} 0\\ 0\\ -m_{i}g_{0} \end{array}\right]$$ (17)

where *m*_*i*_ is the mass of link *i* and *g*_0_ is the gravity constant.

Taking into account all of the joints of the manipulator, the following equation system represents the relationship from Eq. 15

$$\left[\begin{array}{c} \tau_{1}\\ \vdots \\ \tau_{7} \end{array}\right]=\left[\begin{array}{ccc} J_{m,1}^{T} & \cdots & J_{m,7}^{T}\\ \vdots & \ddots & \vdots \\ 0 & 0 & J_{m,7}^{T} \end{array}\right]\left[\begin{array}{c} g_{1}\\ \vdots \\ g_{7} \end{array}\right]$$ (18)

Due to (17) the equation system (18) can be simplified since always only the third entry $J_{m,z,i}^{T}$ of the transposed Jacobian in (16) of link *i* is multiplied by a value not equal to 0. Therefore (18) can be written as

$$\left[\begin{array}{c} \tau_{1}\\ \vdots \\ \tau_{7} \end{array}\right]=\left[\begin{array}{ccc} J_{m,z,1}^{T} & \cdots & J_{m,z,7}^{T}\\ \vdots & \ddots & \vdots \\ 0 & 0 & J_{m,z,7}^{T} \end{array}\right]\left[\begin{array}{c} -m_{1}g_{0}\\ \vdots \\ -m_{7}g_{0} \end{array}\right]$$ (19)

In order to calculate the gravity torques of a robot according to (19) the masses *m*_*i*_ as well as the centers of gravity *r*_*com*,*i*_ of the links, which are used to calculate $J_{m,z,i}^{T}$ according to (16), must be known. Since this is not the case without doing an identification of the robot, we replace the distance *r*_*com*,*i*_ to the center of mass of the link i in (16) by the distance *p*_*i*+1_ to the following joint frames. This distance can be calculated from the geometric robot parameters for any joint configuration. This results in

$$\begin{array}{ll} {\tilde{J}}_{m,i}^{T} & ={\left[z_{j}\times p_{i+1}\right]}^{T}\\ & ={\left[\begin{array}{c} {\tilde{J}}_{m,x,i}^{T}\\ {\tilde{J}}_{m,y,i}^{T}\\ {\tilde{J}}_{m,z,i}^{T} \end{array}\right]}^{T} \end{array}$$ (20)

Moreover we replace the unknown link masses *m*_*i*_ in (19) by variables *w*_*i*_. These variables *w*_*i*_ can be understood as imaginary point masses, which are directly located in the origin of the following joint frame. For example *w*_1_ would be the imaginary mass of link 1, located in the origin of joint 2. Replacing *m*_*i*_ by *w*_*i*_ as well as replacing $J_{m,z,i}^{T}$ by ${\tilde{J}}_{m,z,i}^{T}$ in (19) results in

$$\left[\begin{array}{c} {\tilde{\tau }}_{1}\\ \vdots \\ {\tilde{\tau }}_{7} \end{array}\right]=\left[\begin{array}{ccc} {\tilde{J}}_{m,z,1}^{T} & \cdots & {\tilde{J}}_{m,z,7}^{T}\\ \vdots & \ddots & \vdots \\ 0 & 0 & {\tilde{J}}_{m,z,7}^{T} \end{array}\right]\left[\begin{array}{c} -w_{1}g\\ \vdots \\ -w_{7}g \end{array}\right]$$ (21)

This relationship describes the influence of the gravity force of the imaginary masses on the joint torques of the robot. Our goal is to determine fitting imaginary masses *w*_*i*_, located in the origin of the following joints, which have the same influence on the joint torques as the original masses *m*_*i*_ located in the center of mass *r*_*com*,*i*_ of the link. Then approximated gravity joint torques $\tilde{\tau_{i}}$ can be calculated according to (21) for any joint configuration.

To determine *w*_*i*_, the equation system in (21) must be solved. This can be done in the least-square sense using *N* measurements, where *N* ≫ 7. We selected *N* = 500 for calculating the optimal linear regression solution $x={\left[w_{1},\ldots ,w_{7}\right]}^{T}$ .

### 2.5 Neural Network Model for Wrench Prediction

External forces applied to a certain point of an attached tool lead to corresponding contact wrenches at the end-effector. Our aim was the estimation of these wrenches w^*ecs*^ based on the measured joint torques *τ* ^Δ^, the current robot pose and the distance to the force application point, given in the input vector *I*. This estimation was performed by training a regression model on simulated data for a static case (Section 2.3.1) and retraining it with real training data (Section 2.3.2). We used a dense feed-forward neural network with fully connected layers. The optimal network architecture for our problem has been found by doing a hyperparameter optimization varying the number of layers, the regularizer, the loss function as well as the number of neurons. The neurons were modeled with rectified linear unit (ReLU) activation functions (Nair and Hinton, 2010). The last layer consists of six neurons to predict the wrench vector f^*ecs*^ with linear activation. The activation functions *g*_*ReLU*_ and *g*_*linear*_ are defined as

$$g_{ReLU}\left(x\right)=max\left(0,x\right)$$ (22)

and

$$g_{linear}\left(x\right)=x.$$ (23)

The wrench vector is predicted by propagating the input through the layers of the network (Rumelhart et al. (1986)). The relationship between w^*ecs*^ and the measured joint torques *τ* ^Δ^ is highly dependent on the robot’s current joint configuration as well as the distance to the point of force application $r_{com}^{\;ecs}$ in the end-effector coordinate system. One challenge was the identification of a suitable, unique representation of the robot’s pose. We observed that the joint angles q_*i*_, *i* ∈ {1 … 7} alone were not sufficient. Even by adding the end-effector rotation and position as inputs, the model was not able to learn the problem and did not converge. The high redundancy of the robotic arm, especially the additional seventh DoF, greatly increases the complexity of the function to be learned. Therefore, we additionally fed the translational and rotational part of the homogenous pose matrices Miecs,i∈{1…7} , of the single joints regarding the end-effector frame to the network model. This results in 12 additional parameters per joint, i.e., the nine rotational parameters ${\text{r}}_{i}^{1},\ldots ,{\text{r}}_{i}^{9}$ and three translational parameters ${\text{t}}_{i}^{1},\ldots ,{\text{t}}_{i}^{3}$ for the *i*th joint. We define the vector Mi′0 as the element-wise representation of the pose matrix Mi0 :

Mi′0=ri1,…,ri9,ti1,…,ti3∈R12,i∈1…7. (24)

Hence, the input *I* and the output *O* of the neural network can be written as

$$\begin{array}{cc} I= & \left[{\text{q}}_{1},\ldots ,{\text{q}}_{7},\tau_{1}^{\Delta },\ldots ,\tau_{7}^{\Delta }{,}^{ecs}M_{1}^{\prime },\ldots {,}^{ecs}M_{7}^{\prime },\right.\\ & \;\left.r_{com,x}^{\;ecs},r_{com,y}^{\;ecs},r_{com,z}^{\;ecs}\right]\in R^{101}. \end{array}$$ (25)

$$O=\left[{\text{f}}_{x}^{\;ecs},{\text{f}}_{y}^{\;ecs},{\text{f}}_{z}^{\;ecs},{\text{m}}_{x}^{\;ecs},{\text{m}}_{y}^{\;ecs},{\text{m}}_{z}^{\;ecs}\right]\in R^{6}$$ (26)

The input *I* was normalized to have zero mean and a unified variance of 1 (zero centering). The model was implemented in *Python* using *Keras* (Chollet, 2015) with the *Theano*-Backend (Theano Development Team, 2016).

#### 2.5.1 Optimal Network Architecture

Firstly, a deep feed forward neural network was trained with the idealized simulated *dataset*_*train*,*sim*_. To find an optimal network architecture for our problem of predicting end-effector wrenches, an autonomous hyperparameter optimization was done using the *optuna* toolbox (Akiba et al., 2019) and the LAMB optimizer (You et al., 2019). In Table 3 the static as well as the varied neural network parameters and their ranges are listed. The loss function for training a particular neural network is an optimization parameter and can vary between mean squared error and mean absolute error (see Table 3). In order to compare the different tested network architectures with an equal metric to find the neural network with the highest prediction accuracy, we calculated a separate evaluation error using the root mean squared error. Training was done on four GPUs of a DGX-2. In total 700 different architectures were tried, which needed a computing time of approximately around 19 days. The resulting optimal neural network model with the highest prediction accuracy is named *NN*_*sim*_. The trained model was used on a regular desktop PC, with an Intel core i7 CPU and a nVidia GeForce GTX 1060. One evaluation step takes 21.5 ms.

**TABLE 3:** Hyperparameter optimization - static and dynamic parameters.

Parameter	Type	Value/Value range
Layers	Dynamic	1–20
Neurons	Dynamic	1–1,000
L2 regularization	Dynamic	1*e* ^−20^ − 1*e* ^−3^
Loss	Dynamic	Mean squared error, mean absolute error
Epochs	Static	500
Batch size	Static	6,000
Early stopping patience	Static	1,000

#### 2.5.2 Retraining With Real Training Data

The optimized neural network model *NN*_*sim*_ resulted from a training process only done with simulated data representing an idealized static case. In a real scenario different inaccuracies like dissipative effects and measurement inaccuracies occur. In addition we do not know the exact isolated torques $tau_{j}^{delta}$ generated by the specific calibration weights. Thus we first have to estimate the torques without mounted calibration weight *τ*_0_ using our proposed gravity compensation model from Section 2.4. Afterwards we can calculate $tau_{j}^{delta}$ according to (14).

To take into account the described inaccuracies, the model based on simulated data *NN*_*sim*_ was retrained with the real training dataset *dataset*_*train*, *real*_. This was done on a regular desktop PC, with an Intel core i7 CPU and a nVidia GeForce GTX 1060. The resulting model is named *NN*_*retrain*_.

## 3 Results and Discussion

The goal is to precisely estimate gravity joint torques as well as contact wrenches at the end-effector from given joint positions and torque data. A data-driven method based on linear regression is used to approximate the static gravity torques without any knowledge of the link masses nor the centers of gravity of the links. To estimate the end-effector contact wrench an extensive database, consisting of both simulated and real data, was acquired to develop and evaluate artificial neural network models. For generating real ground truth contact wrenches, ten specially manufactured weights in the range of 0–2 kg (0–20 N) were mounted to the end-effector. By using the constant gravity force and different robot base orientations, a homogeneous representation of contact forces in all directions was realized. Due to various combinations of base orientations, calibration weights and robot poses, the database consists of 330,000 randomized data points. See Section 2.3.2 for more details. For evaluation purposes, the testing datasets (see Section 2.3.3 and Section 2.3.4) were used, which were not included in the training of the neural networks.

Firstly the performance of our proposed gravity compensation model is evaluated in Section 3.1. Afterwards the capabilities of the neural network models to precisely estimate contact wrenches at the end-effector are illustrated (Section 3.2)–even if another robot is used (Section 3.4).

### 3.1 Accuracy of Gravity Compensation Model

To determine the imaginary masses *w*_*i*_ by solving the overdetermined equation system in (21) 500 data points out of the *dataset*_*train*, *real*_ without mounted calibration weights are used. Increasing the number of data points had not shown any significant change of the results. The datapoints are taken in equal parts from the 6 different base orientations. The optimal solution is (values in N):

$$\begin{array}{ll} x & =-g_{0}{\left[\begin{array}{ccccccc} w_{1} & w_{2} & w_{3} & w_{4} & w_{5} & w_{6} & w_{7} \end{array}\right]}^{T}\\ & ={\left[\begin{array}{ccccccc} 0 & 0 & 74.56 & 0 & 47.1 & 0 & 7.85. \end{array}\right]}^{T} \end{array}$$ (27)

By dividing through the negative gravity constant *g*_0_, we get

$$\tilde{x}={\left[\begin{array}{ccccccc} 0 & 0 & 7.6 & 0 & 4.8 & 0 & 0.8 \end{array}\right]}^{T}$$ (28)

for the imaginary masses *w*_*i*_ (values in kg). The result shows, that only the parameters *w*_3_, *w*_5_ and *w*_7_ have values not equal to zero. So in our model the gravity forces acting on the joint torques of the robot are generated only by three imaginary masses. These are located in joint 4 (robot elbow), joint 6 (robot hand) and in the end-effector.

**FIGURE 4:**
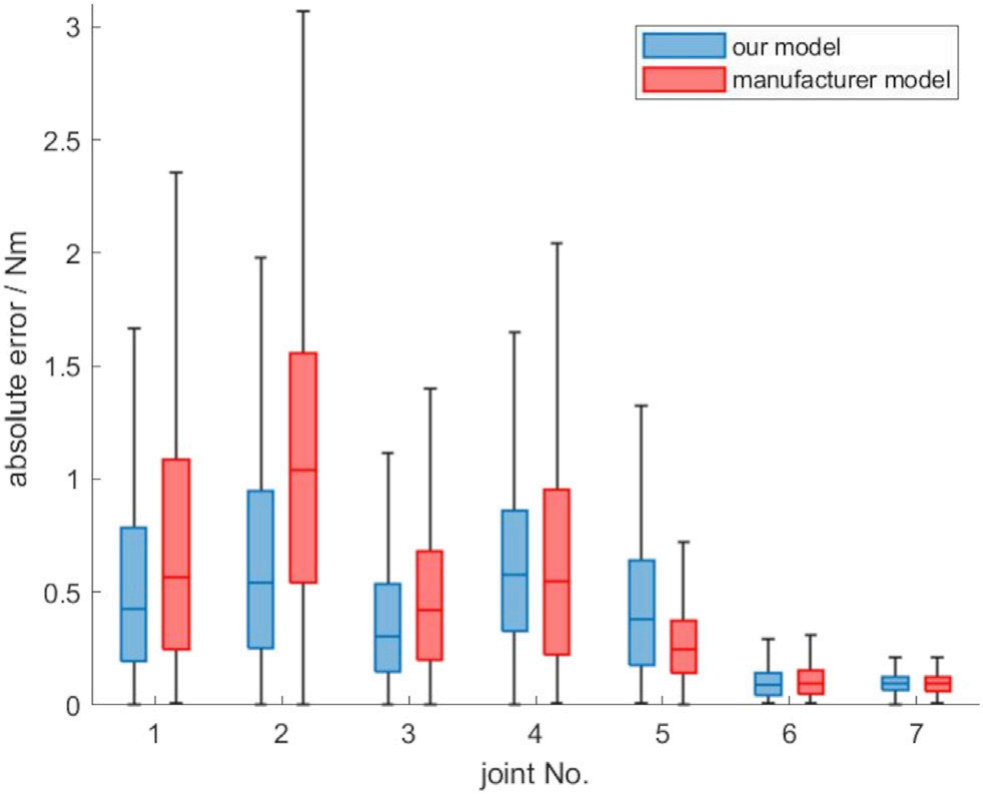
Absolute error of the proposed gravity compensation model compared to the model of the robot manufacturer, evaluated on the *dataset*_*gen*,*dir*_.

The determined imaginary masses *w*_*i*_ are now used to estimate the static gravity torques according to (21). The intra-directionality generalization dataset *dataset*_*gen*,*dir*_. without mounted calibration weights is used for evaluation. Figure 4 shows the absolute errors for the estimated static gravity torques of the joints using our model. The absolute errors of the estimation of the robots integrated PFEM model are also shown in Figure 4. The robot firmware does not directly output the estimated gravity torques, but the measured joint torques as well as the calculated external joint torques. So for a comparision with our model, the external joint torques are used to calculate the absolute error. For a robot without any weight attached to the endeffector, the external joint torques are lower the better the integrated model of the gravity compensation is. Figure 4 shows that our model fits the zero torques in a static experiment slightly better than the integrated model of the robot manufacturer. Especially the median error of the zero torques for the second joint can be reduced from 1.0 to 0.54 N (46%). Moreover the variability could be reduced, except for joint 5. For joint 5 also the median of the prediction error is 34% higher using our model. Our model seems to have difficulties in estimating the torque in joint 5 induced by the masses of the following links 5, 6 and 7. Theoretically there would be two imaginary masses *m*_5_ and *m*_7_ unequal to zero, which are located in joint 6 and the end-effector and thus are taken into account for the computation of the joint torque 5. But considering the equation to approximate $\tilde{\tau_{5}}$ in system (21) the corresponding entry ${\tilde{J}}_{m,z,5}^{T}$ , which is multiplied by *m*_5_, equals zero. So only *w*_7_ has an influence for approximating the gravity torque $\tilde{\text{t}\text{a}{\text{u}}_{5}^{delta}}$ , which explains the prediction inaccuracy.

The obtained results show the applicability of the presented approach to determine static gravity torques of a robot without knowing the inertial parameters of the links. It represents a simple to implement and time-saving option, since only joint torque data of a few randomly generated poses need to be acquired.

### 3.2 Accuracy of Contact Wrench Estimation

For evaluation purposes, the contact forces and moments acting at the end-effector are considered separately. The prediction accuracies of the *NN*_*sim*_, which was only trained with the simulated data for an ideal static case and the *NN*_*retrain*_, which was retrained with the real dataset *dataset*_*train*, *real*_ are compared with the PFEM model.

#### 3.2.1 Optimal Neural Network Architecture

To find the optimal network architecture for the estimation of contact wrenches, an autonomous hyperparameter optimization was done using the *optuna* toolbox (Akiba et al., 2019) and the LAMB optimizer (You et al., 2019). 700 different architectures were tested. The simulated training dataset was divided into training (95%) and validation data (5%). After 514 iterations the lowest validation error was found for a network architecture, consisting of 14 hidden layers with 681 neurons and a L2 regularization of 1.23e-06. The mean absolute error was used as loss function. The created network is referred to as *NN*_*sim*_. On a regular desktop PC, with an Intel core i7 CPU and a nVidia GeForce GTX 1060, one evaluation step takes 21.5 ms. The evaluation time is thus quite high, which can be explained by the large size of the neural network. Depending on the application, this might have to be taken into account when choosing a network architecture. In our current setup assuming a static scenario, where is no or almost no movement of the robot when the force is estimated, the long evaluation time can be handled. Since we expect slow movements, the reaction time is not extremely critical in this context.

#### 3.2.2 Contact Forces

**FIGURE 5:**
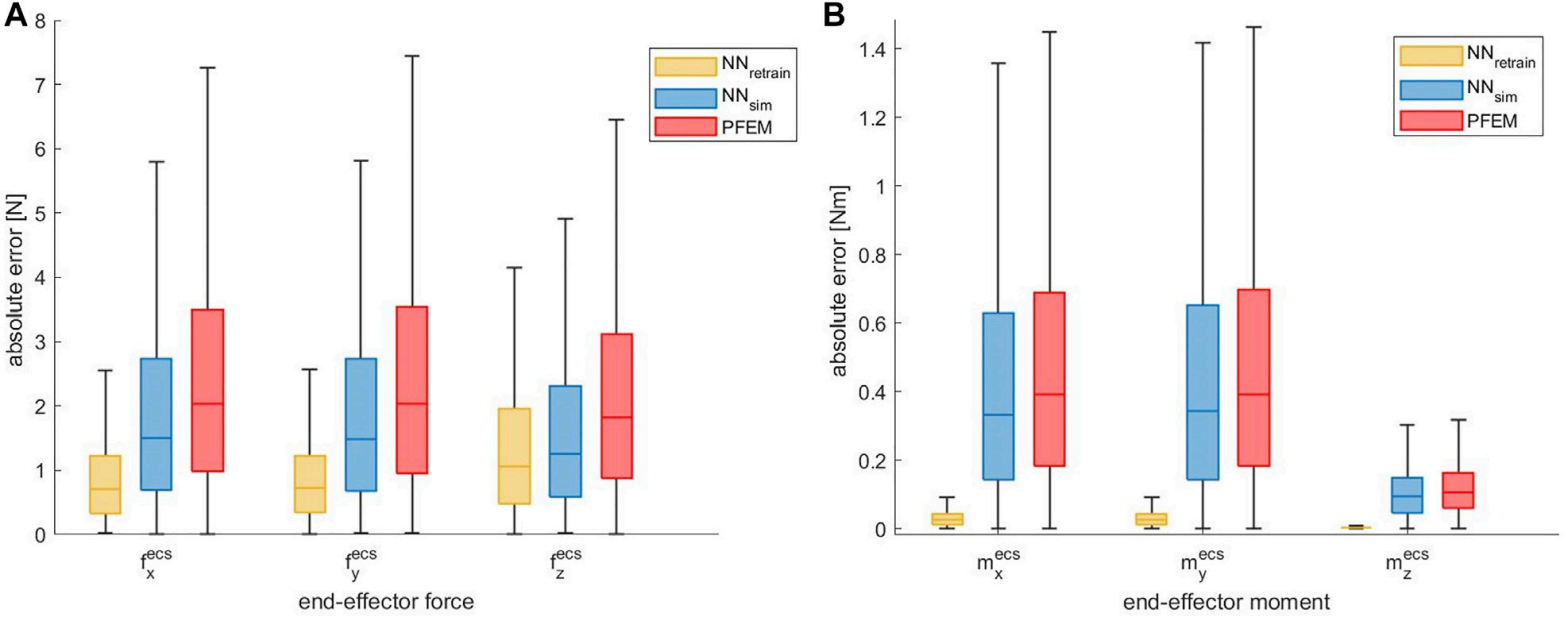
**(A)** Absolute end-effector force error of the ANN approach compared to the PFEM given for each axis in linear scale evaluated on the *dataset*_*gen*,*dir*_. The median error could be largely reduced by the ANN, which was retrained with real robot data. **(B)** Absolute end-effector moment error of the ANN approach compared to the PFEM given for each axis in linear scale evaluated on the *dataset*_*gen*,*dir*_.

Figure 5A shows the overall accuracy of our proposed calibration compared to the PFEM based on all measurements of the *dataset*_*gen*,*rob*_ in a linear scale. The absolute error was calculated for the contact forces f^*ecs*^ for each axis separately. The calculation was based on the absolute difference between the ground truth (see Section 2.3.2) and the output of the trained neural network ${\text{f}}_{\text{a}\text{n}\text{n}}^{\;ecs}$ , as well as the measured contact forces given by the PFEM ${\text{f}}_{\text{f}\text{w}}^{\;ecs}$ . By using the neural network model *NN*_*sim*_ the median absolute error could be reduced by 0.53 N (26.0%) for ${\text{f}}_{x}^{\;ecs}$ , 0.56 N (27.5%) for ${\text{f}}_{y}^{\;ecs}$ and 0.56 N (30.8%) for ${\text{f}}_{z}^{\;ecs}$ . The retrained model *NN*_*retrain*_ shows even better results. Here the median absolute error could be reduced by 1.32 N (65.0%) for ${\text{f}}_{x}^{\;ecs}$ , 1.32 N (64.8%) for ${\text{f}}_{y}^{\;ecs}$ and 0.75 N (41.5%) for ${\text{f}}_{z}^{\;ecs}$ .

#### 3.2.3 Contact Moments

Figure 5B shows the accuracy of our NN models compared to the PFEM. Again the absolute error was calculated for the three axis separately. By using the neural network model *NN*_*sim*_ the median absolute error could be slightly reduced by 0.06 Nm (15.1%) for ${\text{m}}_{x}^{\;ecs}$ , 0.05 Nm (12.7%) for ${\text{m}}_{y}^{\;ecs}$ and 0.01 Nm (9.7%) for ${\text{m}}_{z}^{\;ecs}$ . In contrast, the retrained model *NN*_*retrain*_ leads to a significant reduction of the end-effector moment prediction errors. A reduction of 0.37 Nm (93.4%) for ${\text{m}}_{x}^{\;ecs}$ , 0.37 Nm (93.3%) for ${\text{m}}_{y}^{\;ecs}$ , and 0.10 Nm (96.4%) for ${\text{m}}_{z}^{\;ecs}$ was achieved. The great difference to the predictions of the *NN*_*sim*_ as well as the PFEM could result from our scenario, where the moments acting at the end-effector in the *dataset*_*real*_ are solely generated by the contact-forces and the distance to the force application point. This is due to our experimental setup, where contact wrenches were induced by masses, which were pulled towards the ground by the gravity. Thus no additional external end-effector moments were applied. As the neural network *NN*_*retrain*_ was retrained with these experimental data, it shows a better prediction accuracy for the end-effector moment as long as no additional external moments are applied.

It must also be noted, that our ground truth for the contact moments is based on the center of mass of the metallic rod with the mounted calibration weights. These were determined from an appropriate created CAD model, so small uncertainties can possibly result. Nevertheless it could be shown, that the relationship between end-effector forces, the distance to the force application point and corresponding end-effector moments can be learned and precisely predicted by the neural network. Due to the problematic issues pointed out regarding the contact moment, in the following chapters the accuracy of contact forces acting at the end-effector is investigated in more detail. These are also of higher relevance for our application of precisely estimating the force acting on an ultrasound probe attached to the robot end-effector.

### 3.3 Outlier Reduction

**FIGURE 6:**
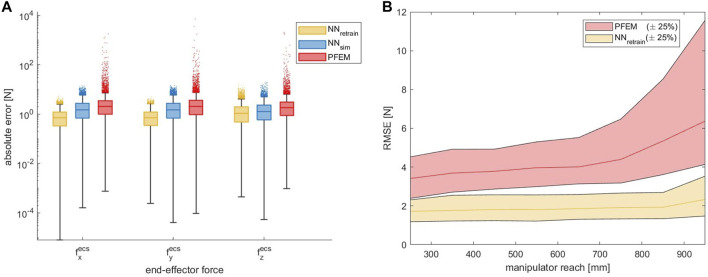
**(A)** Absolute end-effector force error of the ANN approach compared to the PFEM given for each axis in logarithmic scale, evaluated on the *dataset*_*gen*,*dir*_. The outliers could be largely reduced by the ANN approaches. **(B)** Contact force RMSE as a function of increasing manipulator reach in linear scale. A strongly rising error can be observed in the PFEM data from a reach above 700 mm.

As shown in Figure 6A we observed a high number of large outliers in the PFEM data with maximum errors in the range of 10^4^ N. We suspect that these high errors are caused by numerical instabilities of the computational model used by the PFEM near singular configurations. In contrast, our proposed solution provides much more stable results than the integrated model.

In order to analyze the problems of incalculable contact forces at singular positions and numerical instabilities close to these positions in more detail, we looked at the contact force errors as a function of the arm position. To acquire this, the manipulators’ reach was used as a relevant parameter. The reach indicates how far the end-effector is displaced from the shoulder. At maximum reach, the arm is therefore fully extended and in a singular configuration. Figure 6B shows the contact force RMSE as a function of increasing reach. With an increasing reach over 700 mm, the error grows dramatically. In addition, the number and magnitude of outliers also rises massively. In contrast, the ANN data shows much more stable results. The outcome indicates that different arm positions have less impact on the model accuracy and, furthermore, that the model is capable of robustly handling singularities. Clearly, this is counterintuitive: Fundamental mathematical laws render contact forces incalculable at singularities since the matrix **J** from (4) becomes singular. Nevertheless, this does not mean that contact forces in singularities cannot be detected at all, it only means that specific contact forces may not generate torques in all joints, which, in turn, will result in large errors when trying to compute the contact forces. Given that the training data we have collected was generated randomly, it is exceedingly unlikely that the robot was ever moved to an *exactly* singular position, but rather that it often came *close* to one. This means that our model has learned to deal with close-to-singular matrices **J** and, obviously, is capable of adequate compensating. Nevertheless, this does not mean that our model can overcome mathematical impossibilities, it rather means that it is less susceptible to numerical instabilities occurring close to singular positions.

### 3.4 Transfer to Another Robot

**FIGURE 7:**
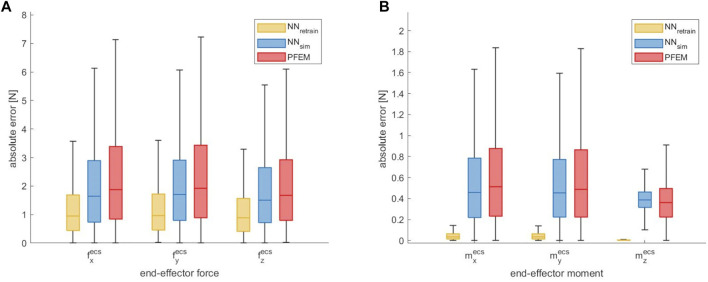
Absolute end-effector force error **(A)** and moment error **(B)** of the ANN approach compared to the PFEM given for each axis in linear scale, evaluated on the transfer data of the *KUKA iiwa 14 R820* *dataset*_*transf*_.

To examine the transferability of our approach to another robot, the accuracy of contact force estimation of the additional testing dataset, which has been acquired with the *KUKA iiwa 14 R820*, is shown in Figure 7A. By using the neural network model *NN*_*sim*_, which was solely trained on simulated data for the robot geometry of the *KUKA iiwa 7 R800*, the median absolute error could be slightly reduced by 0.23 N (12.3%) for ${\text{f}}_{x}^{\;ecs}$ , 0.21 N (10.9%) for ${\text{f}}_{y}^{\;ecs}$ and 0.16 N (9.5%) for ${\text{f}}_{z}^{\;ecs}$ . By using the network *NN*_*retrain*_, which was retrained with the real data of the *KUKA iiwa 7 R800*, the force errors could be reduced by 0.92 N (49.2%) for ${\text{f}}_{x}^{\;ecs}$ , 0.95 N (49.4%) for ${\text{f}}_{y}^{\;ecs}$ and 0.79 N (47.3%) for ${\text{f}}_{z}^{\;ecs}$ . Furthermore the accuracy of the estimation of the contact moments can also be improved (Figure 7B). The retrained model *NN*_*retrain*_ leads to a reduction of the end-effector moment prediction errors. It must be noted, that for the prediction of the contact moments, the same drawbacks as described in Section 3.2.3 arise due to our experimental setup, which require further investigations.

However, it is shown, that the estimation of the contact forces could be improved by our neural network models - even if another robot type is used. Especially the *NN*_*retrain*_ can greatly reduce the force prediction errors. Once again it should be emphasized that no additional training data, neither simulated nor real, of the *KUKA iiwa 14 R820* were used. Retraining on a small real calibration dataset acquired on this different robot would probably reduce the errors even more so that an accuracy similar to the experiments with the *KUKA iiwa 7 R800* in Section 3.2 could be achieved. In this context, it must be noted that our experiments were done on a robot with the same degree of freedom and thus the same size of input data. For a transfer to a robot with a different degree of freedom, the changed size of input data must be taken into account. It might be an option to just replace the input layer of the network for retraining.

## 4 Conclusion

The aim of this work was to precisely determine corresponding contact wrenches at the end-effector from given joint position and torque data of a redundant serial lightweight manipulator (*KUKA LBR iiwa 7 R800*). The results of this work show advantages of our proposed neural learning approach compared to the PFEM. Firstly, the new calibration method can increase the accuracy of end-effector contact forces by 57.2% and the accuracy of end-effector contact moments by 90% compared to the manufacturer’s proprietary force estimation model. Secondly, we show that the calibration stability can be significantly increased with the proposed approach. In contrast to the PFEM, which shows high outliers near singularities, the ANN approach shows robust results. The evaluation indicates that different arm positions do not affect the accuracy and, furthermore, the proposed model can robustly handle singularities.

After the promising results of this work, minor limitations remain. The calibration was performed in a limited force range of 0–20 N, which is significantly below the maximum loads of the manipulator. However, compliant robots are often used in specialized practical applications within limited load ranges. Moreover, our results show good generalization performance, even for estimation of the neural network, which was soley trained with simulated data. Thus using a huge simulation dataset with an increased force range and a small real calibration dataset with larger distances between the calibration weights could be used. In addition we plan to further investigate the transferability of our method model to robots from other manufacturers, which have a more different geometry. In this context, a transfer to a robot with a different degree of freedom and thus an input of a different size must also be considered. A robot specific simulated dataset could be generated easily for any robot whose geometric data are known. Afterwards a small number of robot specific new recorded data points could be acquired and used for retraining and thus the prediction performance could be improved.

## Data Availability

The raw data supporting the conclusion of this article will be made available by the authors, without undue reservation.
